# Design and Characterization of Chitosan Nanoformulations for the Delivery of Antifungal Agents

**DOI:** 10.3390/ijms20153686

**Published:** 2019-07-27

**Authors:** Natalia L. Calvo, Sruthi Sreekumar, Laura A. Svetaz, María C. Lamas, Bruno M. Moerschbacher, Darío Leonardi

**Affiliations:** 1 Instituto de Química Rosario (IQUIR, CONICET-UNR), Suipacha 531, Rosario S2002LRK, Argentina; 2 Área Análisis de Medicamentos, Facultad de Ciencias Bioquímicas y Farmacéuticas, Universidad Nacional de Rosario, Suipacha 531, Rosario S2002LRK, Argentina; 3 Institute for Biology and Biotechnology of Plants (IBBP), Westfälische Wilhelms-Universität Münster, Schlossplatz 8, 48143 Münster, Germany; 4 Área Farmacognosia, Facultad de Ciencias Bioquímicas y Farmacéuticas, Universidad Nacional de Rosario, Suipacha 531, Rosario S2002LRK, Argentina; 5 Área Técnica Farmacéutica, Facultad de Ciencias Bioquímicas y Farmacéuticas, Universidad Nacional de Rosario, Suipacha 531, Rosario S2002LRK, Argentina

**Keywords:** chitosan nanocapsules, drug delivery, anti-fungal, biological activity, vaginal candidiasis, econazole nitrate, tioconazole

## Abstract

Among different Candida species triggering vaginal candidiasis, *Candida albicans* is the most predominant yeast. It is commonly treated using azole drugs such as Tioconazole (TIO) and Econazole (ECO). However, their low water solubility may affect their therapeutic efficiency. Therefore, the aim of this research was to produce a novel chitosan nanocapsule based delivery system comprising of TIO or ECO and to study their suitability in vaginal application. These systems were characterized by their physicochemical properties, encapsulation efficiency, in vitro release, storage stability, cytotoxicity, and in vitro biological activity. Both nanocapsules loaded with TIO (average hydrodynamic size of 146.8 ± 0.8 nm, zeta potential of +24.7 ± 1.1 mV) or ECO (average hydrodynamic size of 127.1 ± 1.5 nm, zeta potential of +33.0 ± 1.0 mV) showed excellent association efficiency (99% for TIO and 87% for ECO). The analysis of size, polydispersity index, and zeta potential of the systems at 4, 25, and 37 °C (over a period of two months) showed the stability of the systems. Finally, the developed nanosystems presented fungicidal activity against *C. albicans* at non-toxic concentrations (studied on model human skin cells). The results obtained from this study are the first step in the development of a pharmaceutical dosage form suitable for the treatment of vaginal candidiasis.

## 1. Introduction

**Figure 1:**
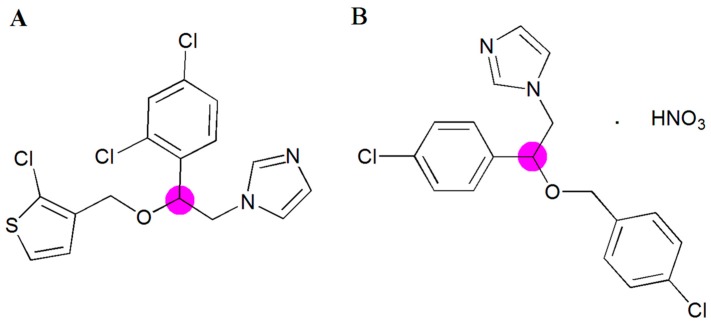
Chemical structure of Tioconazole (**A**) and Econazole nitrate (**B**). ● Chiral center.

*Candida albicans* is an opportunistic fungal pathogen residing in the gastrointestinal and genitourinary tracts as a commensal [1,2]. However, it may act as pathogenic agent causing the infection of the mucous membranes of the vagina, affecting women with both an abnormal and normal immune system. Changes in the immunity of the patients, diabetes, and hormonal status can promote the onset of infection. Among the antifungal agents currently available to treat mucosal candidiasis are Tioconazole (TIO) and Econazole nitrate (ECO) both belonging to the azole family [3]. Azole agents have a common mechanism of action which is based on interfering with biosynthesis of ergosterol and thereby altering the normal functions of the cell membrane, causing cell death [4,5]. TIO (1-[2-[(2-chloro-3-thienyl)methoxyl]-2-(2,4-dichlorophenyl)ethyl]-1*H*-imidazole, Figure 1A) and ECO (1-[2-[(4-chlorophenyl)methoxyl]-2-(2,4-dichlorophenyl)ethyl]-1*H*-imidazole Figure 1B) are highly lipophilic antifungal drugs (log P values of 4.4 [6] and 5.61 [7], respectively) classified into class II (high permeability and low solubility) of the Biopharmaceutical Classification System [8].

These drugs have been found to be effective against *C. albicans* [8,9]; however, their low water solubility may affect their effective concentration in the target site (mucosa) and therefore their therapeutic action. To overcome these problems, different formulations based on cyclodextrins, films, and gels have been developed for the treatment of vaginal candidiasis [8,10,11,12]. Moreover, it has been reported that antifungal resistance continues growing, urging a need for the development of new bioactive compounds and new formulations of antifungals for treatments against Candida infections [4]. In this regard, the development of controlled and targeted drug delivery systems represents one of the main challenges in pharmacological therapy. Particularly, it has been reported that the development of nanocapsules (NC) as drug delivery systems is a useful tool to overcome multidrug resistance [13,14,15,16,17]. The main advantages of NC over traditional formulations are sustained release, incremental drug selectivity and effectiveness, improvement of drug bioavailability, and alleviation of drug toxicity [18].

Nanocapsules are remarkably interesting drug delivery systems, presenting promising therapeutic applications [19]. In this regard, particularly interesting are colloidal NCs consisting of an oily core (e.g., lecithin) surrounded by a hydrophilic biopolymer (e.g., chitosan) because of their promising potential as an effective drug delivery platform for transmucosal administration [20,21,22,23,24,25]. These capsules are unique in terms of their ability to protect poorly water-soluble drugs against chemical or enzymatic degradation [26]. Chitosan and its chemical derivatives have gained particular interest to be used as building blocks for drug delivery nano-formulations in light of their biocompatibility, biodegradability, and mucoadhesivity [27,28,29,30,31]. Formulations based on chitosan present some advantages which are: In situ gelling performance, mucoadhesive properties, and ability to prolong the release of low-molecular-weight compounds to macromolecular drugs [28,32]

Thus, the aim of this study was to develop chitosan NC containing TIO and ECO suitable for vaginal application. The formulated drug delivery systems were fully characterized regarding their physicochemical characteristics such as Z-average particle size, polydispersity index, zeta potential and morphology. In vitro release studies and stability in biological media and simulated vaginal fluid were also performed. Finally, cytotoxicity evaluation and biological activity assay were also carried out.

## 2. Results and Discussion

### 2.1. Physicochemical Properties of Tioconazol and Econazole Nanoformulations

**Figure 2:**
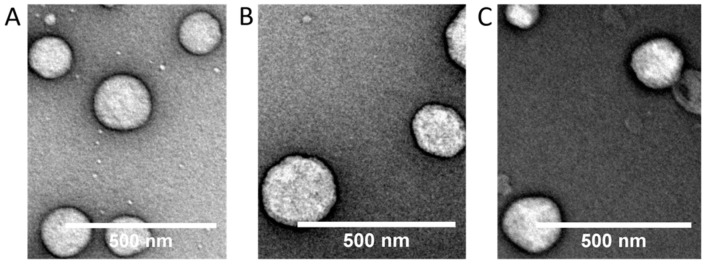
Transmission electron microscopy (TEM) images of nanocapsules (**A**) unloaded (**B**) NC_TIO (**C**) NC_ECO. Scale bar: 500 nm.

Table 1 presents physicochemical parameters and association efficiency of both nanocapsules loaded with TIO (NC_TIO) and loaded with ECO (NC_ECO). The Z-average diameter of each formulation had a narrow range from ∼127 to ∼147 nm, with a low polydispersity index (PdI) fluctuating from ∼0.08 to ∼0.11. The NCs were highly positively charged with zeta potential (ζ) values ranging between +24.7 and +33.0 mV. However, drug-loaded capsules exhibited lower ζ potentials than the unloaded NC, possibly due to the fact that the drugs have nitrogenous atoms in their imidazole group that can be positively charged at the low pH used to dissolve the chitosan. These charged groups can interact with the negatively charged lecithin layer, leaving fewer negative groups of lecithin to interact with the positively charged chitosan, and therefore lowering the net positive charge. Furthermore, a spherical morphology of the loaded and unloaded NC was confirmed using Transmission Electron Microscopy (TEM, Figure 2).

Although the unloaded nanoemulsions (NE) presented a small size (∼139 nm) and a low PdI (∼0.17) with an expected negative ζ potential, the drug-loaded NE aggregated almost instantly and therefore these were not used for further analysis.

The drug-loaded NC displayed a very high association efficiency for ECO (87%) and TIO (99%) as quantified by High-Performance Liquid Chromatography (HPLC).

### 2.2. Stability of NCs

**Figure 3:**
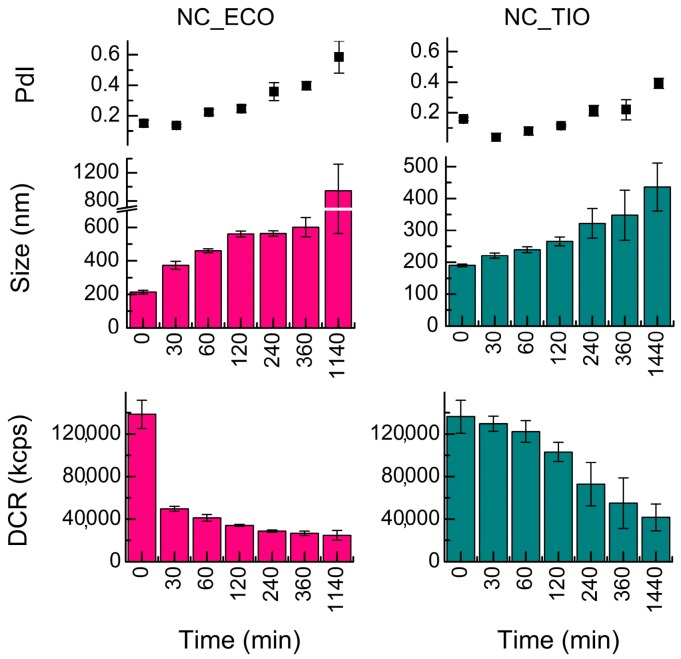
Evolution of the size (Z-average diameter), polydispersity index (PdI), and derived count rate (DCR) with time of drug-loaded nanocapsules (NC) incubated at 37 °C in simulated vaginal fluid (SVF) and acetate buffer. Data shown are means ± SD (*n* = 3).

The colloidal stability [33] of drug-loaded and unloaded (blank) NC over a period of 24 h was determined (Figure 3) in SVF.

Unloaded NCs were the least stable systems in the release media (Figure 3). They showed a PdI of 1 and a size ~2000 nm almost instantly in SVF. Among the drug-loaded NC, TIO-associated capsules showed higher stability against increase in size and PdI when compared to ECO-loaded NC.

### 2.3. Release Assays of TIO- and ECO-Loaded NCs

**Figure 4:**
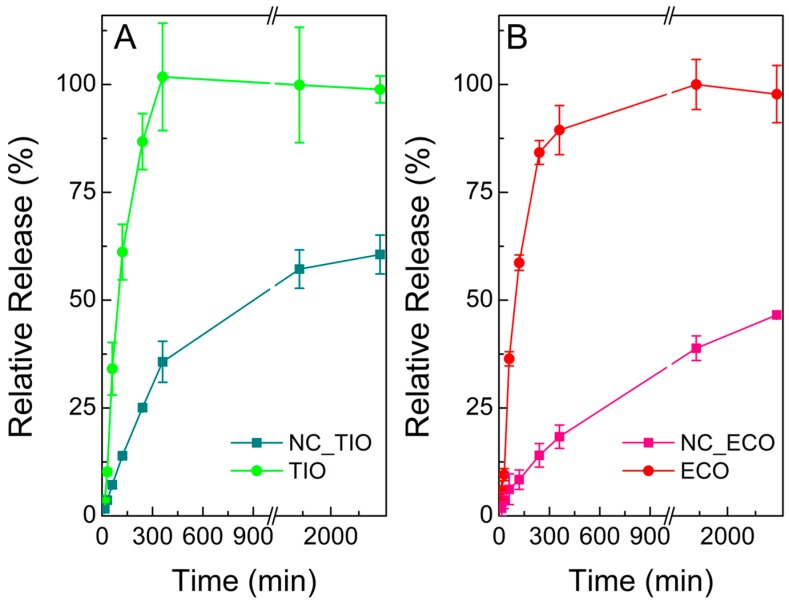
Release curve of (**A**) Tioconazole (TIO) and nanocapsules loaded with TIO (NC_TIO) and (**B**) Econazole (ECO) and nanocapsules loaded with ECO (NC_ECO) in SVF. Data shown are means ± SD (*n* = 3).

The release of both drug-loaded NC was examined in SVF medium (Figure 4). The release of TIO from NC_TIO (Figure 4A) showed a fast profile between 0 and 360 min which slowed down afterwards. The amount of drug released after 360 min was found to be 13.4 ± 1.7 µg/mL, while free TIO reached a concentration of 37.9 ± 4.6 µg/mL. At the end of the experiment (2880 min), 60% of TIO was released from NC_TIO (21.8 ± 1.7 µg/mL) as compared to free TIO (36.9 ± 1.2 µg/mL).

The profile of NC_ECO shows a sustained drug release. The amount of drug released at 2880 min (two days) was found to be 5.6 ± 0.1 µg/mL. This value represents a 46% of drug release with respect to the solution of ECO (11.6 ± 0.8 µg/mL).

Different mathematical models were used to characterize the release kinetics by fitting curves to the release data obtained in SVF. When the full curves were considered, the best fitting model was obtained applying the Weibull function for both formulations (Table 2 and Table 3), which presented the highest values for R^2^-adjusted and model selection criterion, and lowest values for the Akaike Information Criterion. The Weibull model is more useful for comparing the release profiles of matrix type drug delivery [34,35]. The value of the exponent β is an indicator of the mechanism of transport of a drug through the polymer matrix [36]. According to the results, β was <1 for both formulations (0.368 for TIO and 0.493 for ECO). Papadopoulou et al. [36] showed that β-values ≤0.75 indicate Fickian diffusion in either fractal or Euclidian spaces [37]. Additionally, for NC_ECO, the Higuchi model can also be considered as another supporting method [38] as the function also gave reasonably good results.

### 2.4. Storage Stability

**Figure 5:**
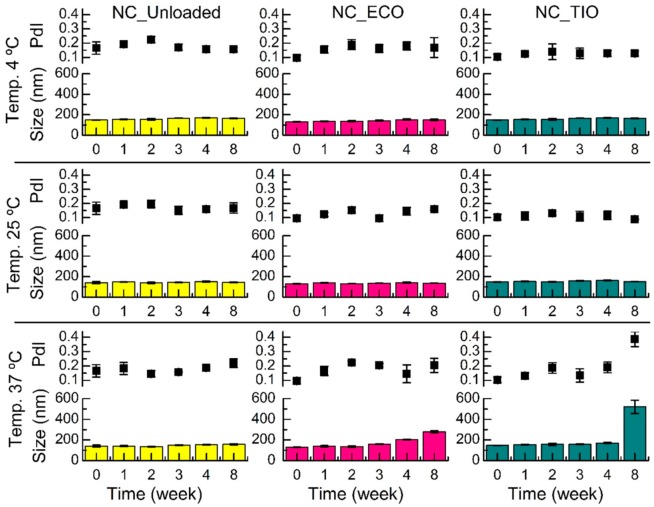
Evolution of size (Z-average diameter) and PdI over time of drug-loaded and unloaded (blank) NC incubated at 4, 25, and 37 °C. Data shown are means ± SD (*n* = 3).

The storage stability of both drug-loaded and unloaded (blank) NC was studied at three temperatures (4, 25, and 37 °C) for eight weeks. The average size and PdI (Figure 5) remained constant for eight weeks in all the systems at 4 and 25 °C, in agreement with previous studies on nanocapsules [39]. However, by the end of eight weeks at 37 °C, the NC loaded with TIO presented a larger PdI and increased their size about 3.5-fold compared to time point zero. This increase might be related to an agglomeration at higher temperature.

**Figure 6:**
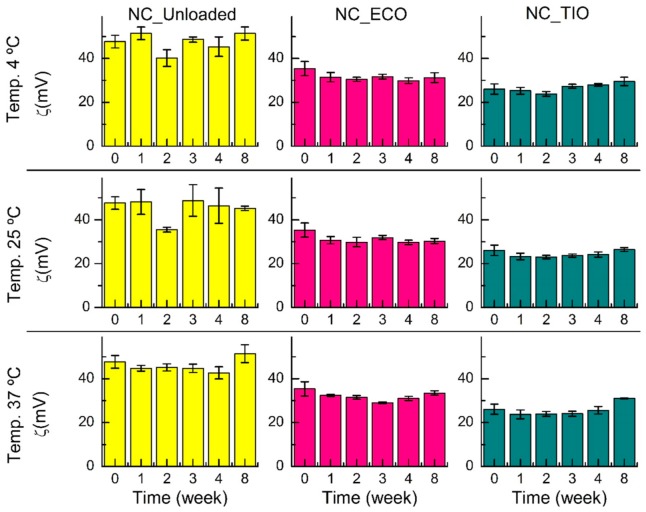
Evolution of the **ζ** potential over time of drug-loaded and unloaded (blank) NC incubated at 4, 25, and 37 °C. Data shown are means ± SD (*n* = 3).

Although it has been reported that loaded chitosan NC may decrease their zeta potential during storage [40], the ζ potential of the systems obtained in this research (Figure 6) remained constant for eight weeks at the three temperatures, which shows the colloidal stability of the developed systems. The result obtained at 37 °C for NC_TIO could be explained by taking into account that an increasing temperature leads to a decrease in the dynamic viscosity. According to the Stokes–Einstein equation, an increase in temperature and a decrease in dynamic viscosity results in an increase of the diffusion constant. A higher diffusion constant leads to faster diffusion of the particles. Having higher kinetic energy, the repulsion between the particles can be overcome more easily, which results in particle aggregation [41]. Flocculation can be generally defined as the aggregation of droplets to give 3-D clusters without coalescence occurring. Importantly, all droplets maintain their own integrity and remain as totally separate entities. This results when there is a weak net attraction between droplets [42]. Thus, flocculation without coalescence could explain the increase in the size of the 3-D cluster observed without modification of the zeta potential.

### 2.5. In Vitro Cytotoxicity

The influence of different concentrations of TIO- and ECO-loaded chitosan NCs on the viability of human keratinocyte cell line (HaCaT) was studied by means of the 3-(4,5-dimethylthiazol-2-yl)-2,5-diphenyltetrazolium bromide (MTT) assay. This is a colorimetric assay used to assess cell viability that relies on mitochondrial metabolic activity. NAD(P)H-dependent cellular oxidoreductase enzymes can reduce colorless MTT to purple formazan. The metabolic activity of a cell is, thus, directly proportional to the absorbance of the formazan crystals. HaCaT cells were used as a model cell line as they are epithelial cells derived from adult human skin that exhibit normal differentiation capacity [43]. Prior to the cytotoxicity studies, drug-loaded NCs were first tested for their stability in the culture media (Figure S2).

**Figure 7:**
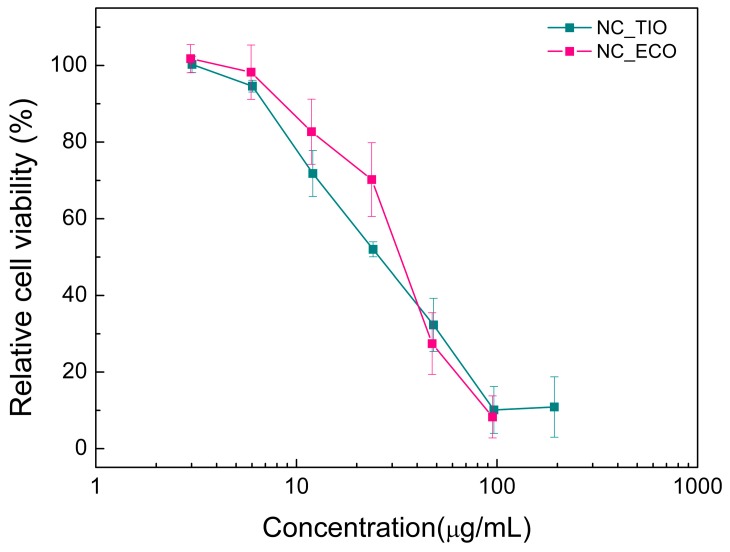
In vitro cytotoxicity of drug-loaded chitosan NC on human keratinocyte cell line (HaCaT) cells in 96-well plates determined using the MTT assay after 24 h. Eight replicates were performed for each treatment. Data shown are means ± SD (*n* = 3).

HaCaT cells were incubated with drug-loaded NC at a concentration of 95^−3^ and 194^−3^ µg/mL for ECO and TIO, respectively. Additionally, seven serially diluted (comparable to the dilution made for drug-loaded capsules) unloaded nanocapsules were also tested as controls to confirm their non-toxic effect. After an incubation period of 24 h, these cells were examined for their viability. Figure 7 summarizes the results of the relative cell viability of HaCaT cells treated with TIO and ECO-loaded chitosan NC. In both treatments, a relative cell viability ≥80% was observed at concentrations ≤12 µg/mL. However, viability of the cells was reduced with increasing concentrations up to ~195 µg/mL for TIO and up to ~95 µg/mL for ECO. This indicated that both drugs had similar effects, presenting very low cytotoxicity on HaCaT cells at the lower concentrations tested.

### 2.6. Biological Activity against Candida Albicans

#### 2.6.1. Antifungal Susceptibility Testing 

Antifungal susceptibility test of drug-loaded and unloaded NC was carried out and compared to both pure drug solutions in dimethyl sulfoxide (DMSO). The Minimum inhibitory concentrations (MICs) and the minimum fungicidal concentration (MFCs) against *C. albicans* are summarized in Table 4.

Chitosan NCs (NC_TIO, NC_ECO) showed activity against *C. albicans* and the MIC values were comparable to that of the drug solutions in DMSO. Thus, it can be seen that the process of nano-encapsulation maintained the activity of both drugs. Similar to our observation, retention of antifungal activity against *C. albicans* by ε-caprolactone NC and NE containing TIO has been reported previously by Ribeiro et al. [44] by using the halo inhibition test. However, it is remarkable to mention that antifungal solutions are discouraged for vaginal applications due to their poor retention in the vaginal tract by the tract’s self-cleansing action [45].

The MFC of the loaded NCs after 24 h of growth was found to be 48.5 µg/mL, while a lower MFC value (3.03 µg/mL) was determined after 48 h. This result can be attributed to the release of drugs into the media after 24 h before transferring them onto the SDA plates. Additionally, control experiments with unloaded chitosan NC and chitosan solution were also carried out. These experiments confirmed no interference of these excipients.

#### 2.6.2. Time-to-Kill

**Figure 8:**
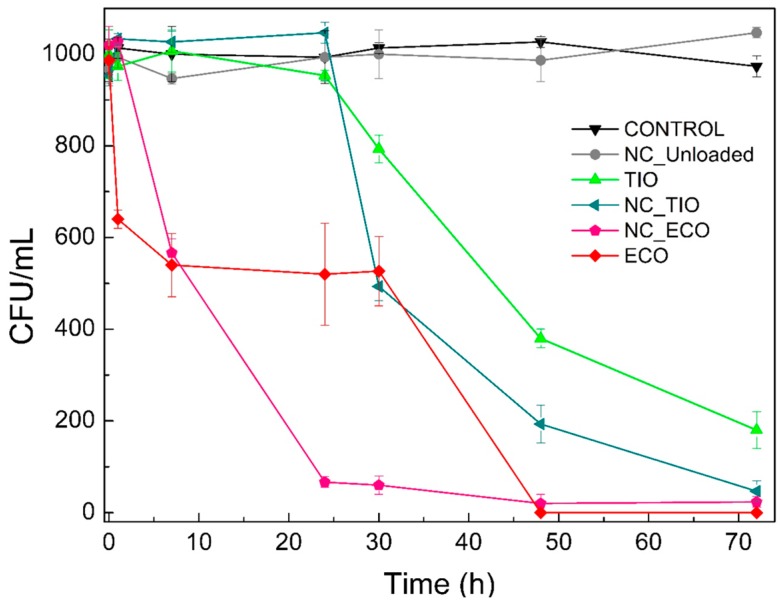
*C. albicans* surviving at each time point when treated with different formulations. Data shown are means ± SD (*n* = 3).

These studies were performed to understand the exposure time required to kill a standardized *C. albicans* inoculum and the results obtained for the drug activity was plotted as colony forming units (CFU)/mL versus time (Figure 8). All samples were tested at 12 µg/mL and, in case of the unloaded NCs, a dilution proportional to that used for NC_ECO was employed. Among the test samples, clearly, the NC_ECO and ECO in DMSO showed higher efficiency when compared to the TIO in DMSO or NC_TIO.

An apparent ~90% decrease in the CFU/mL value within the first 24 h of treatment was observed for NC_ECO. However, at a similar time point, ECO solution in DMSO only reduced CFUs by ~50%. However, by the end of the experiment, both the solution and the NC loaded with ECO were able to completely eradicate a *C. albicans* culture. On the other hand, NC_TIO showed a better time-to-kill profile than TIO solution, the NC completely kill the fungus by 72 h. The NC_TIO samples led to a steep decrease in the CFU/mL at 30 h (~50%) whereas the TIO solution required a longer time to reach a similar value and did not achieve a complete wipe-out of the fungus even after 72 h.

At this point, it must be noted that the concentration at which the drug-loaded NC showed fungicidal activity was well within the non-toxic concentration range as seen from our previous section. These results further strengthen our argument on the use of drug-loaded NCs for the application of vaginal candidiasis.

## 3. Materials and Methods

### 3.1. Chemicals

ECO and TIO of pharmaceutical grade were purchased from AlfaAesar (Kandel, Germany) and Saporiti (Buenos Aires, Argentina), respectively. Biomedical grade chitosan was purchased from Heppe Medical Chitosan (Halle-Saale, Germany), Code Nr. HMC 70/5 (Batch No. 212-170614-01), degree of acetylation and molecular weight determined by ^1^H-NMR and HPSECRID-MALLS were 20% and 29 kDa, respectively. Lecithin (a phosphatidylcholine enriched fraction of soybean lecithin) was a kind gift from Cargill (Epikuron 145V, Cargill Deutschland GmbH & Co. KG, Hamburg, Germany); Miglyol 812 N was obtained from Sasol GmbH (Witten, Germany). Ultrapure double-distilled water was used for the preparation of aqueous solutions. All other chemicals were of analytical grade.

### 3.2. Nanoformulation Preparation

NCs were obtained by the solvent displacement technique first described by Calvo et al. [21]. Briefly, an organic phase was formed by dissolving 40 mg of lecithin in 1 mL of ethanol, followed by the addition of 125 µL of Miglyol 812 (a neutral oil formed by esters of caprylic and capric fatty acids and glycerol) containing the lipophilic drug molecules, and adding ethanol up to 10 mL. The 10 mL of organic phase was poured into 20 mL of the aqueous phase composed of a chitosan solution (0.5 mg/mL in water, dissolved with 1 M HCl in 5% stoichiometrical excess) in case of NC, or into 20 mL of water in case of NE. Both NCs and NE were formed spontaneously by diffusion of organic solvents. Finally, ethanol and part of the water were evaporated at 50 °C under vacuum on an R-210 Rotavapor (Büchi Labortechnik GmbH, Essen, Germany) to reduce the volume of the formulations to 10 mL.

### 3.3. Reverse-Phase High-Performance Liquid Chromatography (RP-HPLC with UV Detection)

RP-HPLC-UV was carried out using a Jasco HPLC system (Jasco GmbH, Gross-Umstadt, Germany) comprising a three-line degasser (DG-2080-53), a ternary gradient unit (LG-2080-02S), a semi-micro HPLC pump (PU-2085Plus), an autosampler (X-LC™ 3159AS), an intelligent column thermostat (CO-2060 Plus) equipped with a Kinetex C-18 reversed phase column (2.6 µm, C18, 50 × 2.1 mm, S/N 539947-37, Phenomenex, Torrance, USA) thermostatted at 50.0 °C, and a UV/Vis detector (X-L™ 3075UV). A mixture of water and acetonitrile (10:90) for TIO, or a mixture of water, acetic acid, and acetonitrile (9:1:90) for ECO was used as the mobile phase in isocratic mode with a flow rate 0.2 mL/min. TIO was detected at λ = 219 nm and ECO at λ = 230 nm. The working range of the method (Figure S1) was 2.0–55.0 µg/mL for TIO (*y* = 0.1037*x*; *R*^2^ = 0.9988), and 0.9–27.3 µg/mL for ECO (*y* = 0.0778*x*; *R*^2^ = 0.9976). The samples were first filtered through 0.45 µm membrane filter before being injected in triplicates.

### 3.4. Association Efficiency

Encapsulation efficiency for each drug was determined using the HPLC methods described above. Briefly, aliquots of the NC (500 µL) were pipetted into Vivaspin 500 ultrafiltration spin columns with Molecular Weight Cut-Off (MWCO) of 3000 dalton (Sartorius AG, Göttingen, Germany) and centrifuged (Mikro 220 R, Hettich GmbH & Co. KG, Tuttlingen, Germany) at 4000× *g* for 1 h at 20 °C. The resulting supernatant was used to calculate the encapsulation efficiency as the difference between the total amount of drug added in the formulation and the amount present in the supernatant (conc. drug unloaded). The Drug Association Efficiency (AE) was calculated using Equation (1).

(1) $$\mathrm{Drug}\;\mathrm{AE}\;\left(\%\right)\;=\;\frac{\left(Conc.drug\;total\;-\;Conc.drug\;unloaded\right)\;}{Conc.drug\;total}\;\times \;100$$

### 3.5. Physicochemical Characterization

The average size and size polydispersity of the NC were determined using dynamic light scattering with non-invasive back scattering (DLS-NIBS) with a measurement angle of 173°. The zeta potential was measured by mixed laser Doppler velocimetry and phase analysis light scattering (M3–PALS). A Malvern Zeta-sizer NanoZS (Malvern Instruments Ltd., Worcestershire, UK) fitted with a red laser light (λ = 632.8 nm) was used for both methods. The samples were diluted 1:100 in water before measurement carried out in triplicates. The zeta-sizer software (v 7.11) was used to acquire and evaluate the results.

### 3.6. Transmission Electron Microscopy

The ultrastructure of the NC was investigated using TEM. Equal amounts of samples were mixed with uranyl acetate solution (negative staining, 1%, w/v). Samples (8 µL) were placed onto a copper grid covered with Formvar^®^ film and the excess of liquid was removed with the aid of a filter paper. The analyses were performed using JEM-1400 TEM (JEOL, Peabody, MA, USA) operated at 100 kV and captured on AMT 1K CCD using AMTV602 software.

### 3.7. Colloidal Stability in Media

The colloidal stability of NC was investigated in simulated vaginal fluid [46] (SVF, pH 4.3), acetate buffer (pH 4.5), and Dulbecco’s Modified Eagle Medium (DMEM) media supplemented with 10% fetal bovine serum, 1% l-glutamine, and 1% penicillin–streptomycin (10,000 units penicillin and 10,000 units streptomycin in 0.9% NaCl). Stability of NCs was evaluated in terms of evolution of particle size distribution over time (up to ∼24 h) at 37 °C using DLS as described above.

### 3.8. Storage Stability of NCs

Aliquots of loaded and unloaded NC were maintained at different temperatures (4, 25, and 37 °C) in sealed tubes. Particle size and zeta potential of the NC were monitored for a period of two months.

These chitosan-based formulations were also assessed throughout this period for the presence of macroscopic changes such as presence of aggregates, cream formation, flocculation, coalescence, or even changes in color.

### 3.9. In Vitro Release Assay

Release assays were performed in dialysis tubes (Pure-a-lyzer Maxi 0.1–3.0 mL, Mw cut-off = 3 kDa, Sigma-Aldrich GmbH, Steinheim, Germany) containing 800 µL of the NCs and placed in a glass beaker containing 14.2 mL SVF or acetate buffer with 30% of ethanol previously maintained at 37 °C.

At different time points (0, 15, 30, 60, 120, 240, 360, 1440, 2880 min), 500 µL aliquots of the medium were removed and replaced by the same volume of medium. The drug content of the aliquots was determined using HPLC as described above.

Stock standard solutions of TIO (7.5 mg/mL) and ECO (6.8 mg/mL) were prepared by dissolving accurately weighed drug amounts in acetonitrile. Working solutions (1.95 mg/mL for TIO and 0.93 mg/mL for ECO) were prepared by transferring appropriate volumes of the stock solutions into separate volumetric flasks (1 mL) and diluting with the release media. Different release models (explanation of all models in Supplementary Information) were applied to understand the release kinetics of the drug-loaded NC and the best model was chosen based on the goodness of fit. Kinetic modeling was performed using DDSolver Excel Add-in software [47].

### 3.10. Cell Culture

HaCaT used as model cell line in this study was obtained from the dermatological clinic at the University Hospital in Münster, Germany; these cells were treated with drug-loaded NC and tested for cell viability. The cells were cultured using DMEM supplemented with 10% fetal bovine serum, 1% l-glutamine (200 mM), and 1% penicillin–streptomycin (10,000 units penicillin and 10,000 units streptomycin in 0.9% NaCl) in 75 cm^2^ flasks. The cultures were kept in an incubator set to 5% CO_2_ and 37 °C (Sanyo MCO-19AIC, Panasonic Biomedical Sales Europe BV, AZ Etten-Leur, The Netherlands).

### 3.11. Cell Viability

The cytotoxicity of NC loaded with drugs was studied using the MTT assay. Initially 100 µL of HaCaT cell suspension was seeded into a 96-well tissue culture plate at ~10^4^ cells per well and incubated for 24 h to enable attachment of the cells to the surface of the wells. Next, incubated cells were washed twice with PBS and samples at varying concentrations were added into the plates and incubated for another 24 h. Later, samples were removed and replaced with 100 µL media containing 25 µL of MTT solution (5 mg/mL in PBS). The plate was incubated for 3 h and the medium was replaced with 100 µL of DMSO and shaken for 10 min at 300 rpm. Absorbance was measured at λ = 570 nm in a microplate reader using a UV/Vis-spectrophotometer (Thermo Fisher Scientific Multiscan GO 60, Waltham, MA, USA). Relative viability was calculated by dividing the mean value of absorbance of the treatment by the mean absorbance of the negative control, in this case the untreated cells. Triton X-100, 4% in PBS, (Sigma Aldrich, St. Louis, MO, USA) was used as a positive control.

### 3.12. Biological Activity against Candida Albicans

#### 3.12.1. Strains and Culture Conditions

*C. albicans* ATCC 10231 (ATCC = American Type Culture Collection) was grown on Sabouraud-chloramphenicol agar for 48 h at 30 °C and maintained on Sabouraud-dextrose agar (SDA) at 30 °C (Laboratorios Britania, Buenos Aires, Argentina). The fungal inoculum was obtained according to the reported procedures [48] and adjusted to 1–5 × 10^3^ CFU/mL.

#### 3.12.2. Antifungal Susceptibility Testing

MIC of the NC or pure compounds were determined by broth microdilution techniques M27-A3 [48] using Roswell Park Memorial Institute (RPMI)-1640 medium (Sigma–Aldrich, St. Louis, MO, USA). Microtiter trays were incubated in a moist dark chamber at 30 °C for 24 h and then they were read spectrophotometrically with the aid of a VERSA Max microplate reader (Molecular Devices, San José, California, USA). For the assay, pure powder of ECO or TIO was first dissolved in DMSO and further diluted with RPMI medium. The dilutions were made such that in a final volume of 200 µL (≤2% DMSO) of the treatments, a concentration range of 97–0.09 and 194–0.19 µg/mL for ECO and TIO, respectively, was reached. The loaded and unloaded NCs were also diluted in RPMI media in the same proportion before using them for the experiments.

A volume of 100 µL inoculum suspension was added to each well to a final titer of 1–5 × 10^3^ CFU/mL. A growth control well (containing medium, inoculum, and the same amount of DMSO as used in the test-wells) and a sterility control well (sample, medium, and sterile water instead of inoculum) were included. MIC was defined as the lowest concentration of a treatment that results in 100% inhibition of fungal growth.

MFC was determined by subculturing aliquots of 5 µL from wells without visible growth in SDA. The plates were incubated for 24 h at 30 °C (MFC 24 h). Additionally, MFC 48 h was evaluated (the release of the drug was allowed from the systems for 24 h before taking the 5 µL aliquot). The MFC was defined as the lowest concentration of each treatment where 99.9% of the final inoculum is killed in the SDA plates [49]. Each treatment was carried out in triplicates.

#### 3.12.3. Time-to-Kill

*C. albicans* ATCC 10231 was cultured in SDA for 24 h before testing. The inoculum was prepared by suspending five distinct colonies in sterile distilled water and shaking on a vortex mixer for 15 s. The cell suspension was adjusted to a turbidity of a 0.5 McFarland standard (approximately 1–5 × 10^3^ CFU/mL). The test systems included NC_TIO, NC_ECO, unloaded (at the same dilution as NC_ECO), and the respective drugs dissolved in DMSO as controls. All samples were introduced into a 5 mL of inoculum such that the final concentration of the test and the control samples was 12 µg/mL. The suspensions were mixed for 20 s with a vortex mixer, and samples (0.05 mL) were taken at 0, 1, 7, 24, 30, 48, and 72 h, then serially diluted before spreading onto SDA. The plates were incubated for 24 h and the viable colonies were evaluated. The time-to-kill curves were constructed by plotting the mean CFU/mL surviving at each time point for every test or control sample from three independent experiments.

## 4. Conclusions

In the present study, a new chitosan-coated nanocapsule carrier was developed for the delivery of Tioconazole and Econazole. These systems were characterized for their physicochemical properties. The loaded and unloaded nanocapsules showed an average hydrodynamic size of 127.1–146.8 nm, a PdI lower than 0.107, and a zeta potential between +24.7 and +46.8 mV. The encapsulation efficiency was higher than 87% for both systems, and the release data obtained in SVF followed a Weibull function for both formulations.

All systems were stable in terms of size and zeta potential over the time-course of two months at 4, 25, and 37 °C, only the zeta potential of NC_TIO was slightly increased in the last week of the study at 37 °C. Finally, the developed systems presented fungicidal activity against *C. albicans* at non-cytotoxic concentrations for HaCaT. Hence, these systems present a promising strategy for local TIO or ECO delivery, which might be used to load films with these antifungal nanocapsules as a pharmaceutical dosage form suitable for the treatment of vaginal candidiasis. This promising film would combine the properties of the film [8] with the advantages associated with nanocapsules, such as the control of the drug release [50,51].

## Figures and Tables

**Table 1:** Characteristics of formulations (mean ± SD, *n* = 3).

Sample	Nanoemulsions			Nanocapsules				
	Size (nm)	PdI ^a^	ζ (mV) ^b^		Size (nm)	PdI ^a^	ζ (mV)	Drug AE (%) ^c^
Unloaded	139.0 ± 3.8	0.170 ± 0.028	−42.0 ± 4.5		131.7 ± 0.5	0.103 ± 0.004	+46.0 ± 0.7	-
ECO 2 mM	1312 ± 131	0.763 ± 0.06	+1.48 ± 1.6		127.1 ± 1.5	0.107 ± 0.017	+33.0 ± 1.0	87.2 ± 6.1
TIO 5 mM	2215 ± 1348	0.9 ± 0.173	−5.19 ± 1.3		146.8 ± 0.8	0.079 ± 0.019	+24.7 ± 1.1	99.0 ± 0.8

^a^ Polydispersity Index. ^b^ Zeta potential. ^c^ Drug Association Efficiency (%) = [(Conc. drug_total_ − Conc. drug_unloaded_) / (Conc. drug_total_)] × 100.

**Table 2:** Kinetic parameters obtained after fitting curves for different release models to the release data from the NC_TIO.

Formulation	Model	Parameters ^a^		R^2^adj	AIC	MSC
NC_TIO	Zero-order	*K_0_*	0.511	0.6047	54.7691	0.6292
	Higuchi	*K_H_*	3.282	0.9247	38.1915	2.2870
	First-order	*K_1_*	0.006	0.6418	53.7831	0.7278
	Korsmeyer-Peppas	*K_KP_*	4.230	0.9334	37.7848	2.3276
		*n*	0.421			
	Hixson-Crowell	*K_HC_*	0.002	0.6295	54.1226	0.6939
	Weibull	***α***	**17.652**	**0.9639**	**32.3242**	**2.8737**
		***β***	**0.368**			
		***T_1_***	**0.487**			

Numbers in bold indicate that these values are the most relevant. ^a^ *K_0_* zero order release constant. *K_H_* Higuchi release constant. *K_1_* first order release constant. *K_KP_* Korsmeyer-Peppas release constant. *n* diffusional exponent. *K_Hc_* Hixson-Crowell release constant. α scaling parameter. β shape parameter. *T_1_* location parameter.

**Table 3:** Kinetic parameters obtained after fitting the release data from the NC_ECO to different release models.

Formulation	Model	Parameters ^a^		R^2^adj	AIC	MSC
NC_ECO	Zero-order	*K_0_*	0.277	0.7961	35.7050	1.3082
	Higuchi	***K_H_***	**1.717**	**0.9853**	**9.3942**	**3.9393**
	First-order	*K_1_*	0.003	0.8126	34.8618	1.3925
	Korsmeyer-Peppas	*K_KP_*	1.681	0.9836	11.3412	3.7446
		*n*	0.506			
	Hixson-Crowell	*K_HC_*	0.001	0.8072	35.1482	1.3639
	Weibull	***α***	**53.377**	**0.9879**	**8.9542**	**3.9833**
		***β***	**0.493**			
		***T_1_***	**0.222**			

Numbers in bold indicate that these values are the most relevant.

**Table 4:** MIC and MFC (µg/mL) of the systems against *C. albicans.*

Sample	MIC (24 h)	MFC (24 h)	MFC (48 h)
TIO	1.52	24.25	6.06
NC_TIO	1.52	48.50	3.03
ECO	3.03	24.25	3.03
NC_ECO	3.03	48.50	3.03

MIC = Minimum Inhibitory Concentration; MFC = Minimum Fungicidal Concentration.

## Supplementary Materials

Supplementary materials can be found at https://www.mdpi.com/1422-0067/20/15/3686/s1.
