# Supplementary material for: Design and Characterization of Chitosan Nanoformulations for the Delivery of Antifungal Agents
Source: Int J Mol Sci. 2019 Jul 27;20(15):3686. doi: 10.3390/ijms20153686 (PMC6695956; doi:10.3390/ijms20153686)
Supplement: Supplementary file 1 [file ijms-20-03686-s001.pdf]

Supplementary Information

DESIGN AND CHARACTERIZATION OF CHITOSAN NANOFORMULATIONS FOR THE DELIVERY OF ANTIFUNGAL AGENTS

Natalia L.Calvo[a,b,+];Sruthi Sreekumar [c,+]; Laura A. Svetaz[d]; María C. Lamas[a,e]; Bruno M. Moerschbacher*[,c]; Darío Leonardi*[,a,e]

[a]*Instituto de Química Rosario (IQUIR, CONICET-UNR), Suipacha 531, Rosario (S2002LRK), Argentina.*

[b] *Área Análisis de Medicamentos, Facultad de Ciencias Bioquímicas y Farmacéuticas, Universidad Nacional de Rosario, Suipacha 531, Rosario (S2002LRK), Argentina.*

[c] *Institute for Biology and Biotechnology of Plants (IBBP), Westfälische Wilhelms-Universität Münster, Schlossplatz 8, 48143 Münster, Germany.*

[d]*Área Farmacognosia, Facultad de CienciasBioquímicas y Farmacéuticas, Universidad Nacional de Rosario, Suipacha 531, Rosario (S2002LRK), Argentina.*

[e] *ÁreaTécnicaFarmacéutica, Facultad de CienciasBioquímicas y Farmacéuticas, Universidad Nacional de Rosario, Suipacha 531, Rosario (S2002LRK), Argentina.*

[+]Natalia L. Calvo and Sruthi Sreekumar contributed equally to this work.

* Corresponding Authors: leonardi@iquir-conicet.gov.ar (D. Leonardi), moersch@uni-muenster.de (B.M.Moerschbacher).

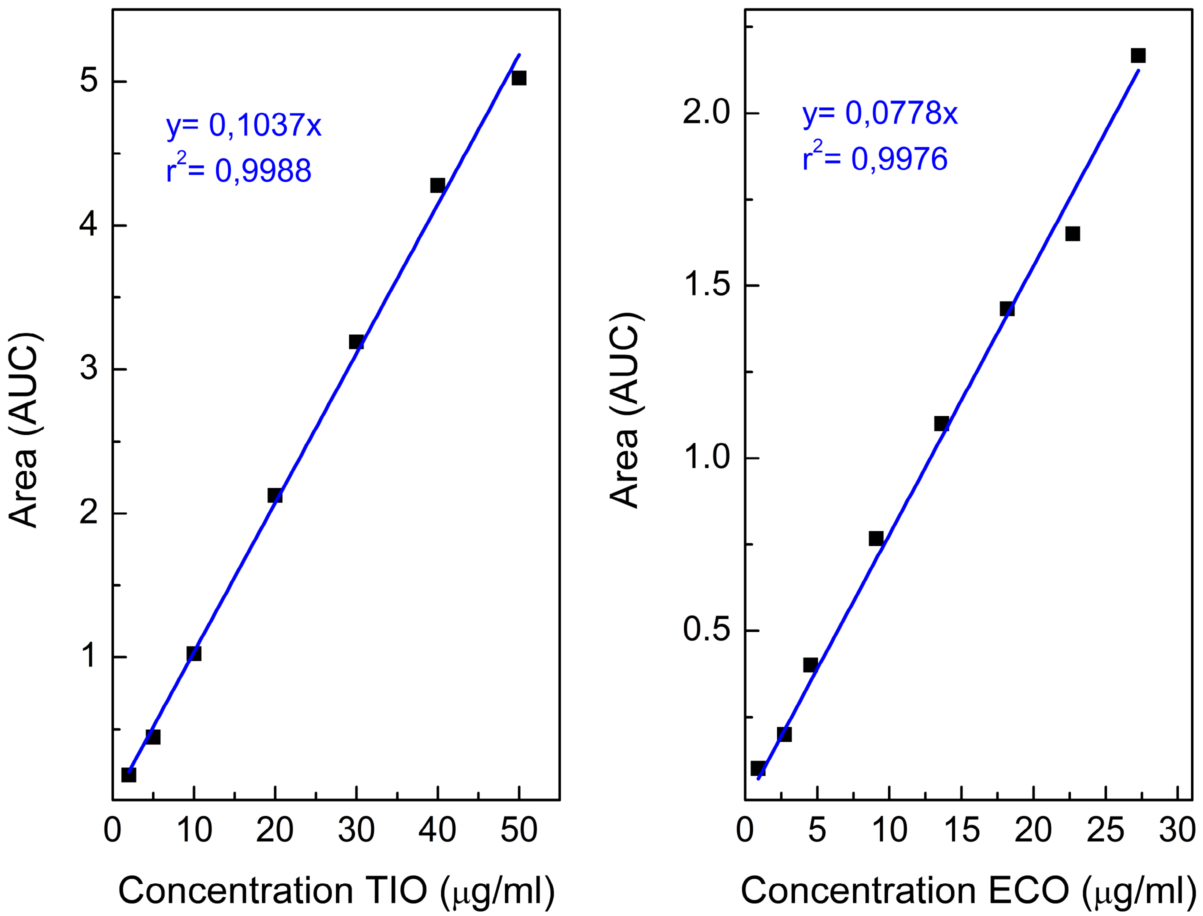

Figure S1. Calibration curve HPLC.

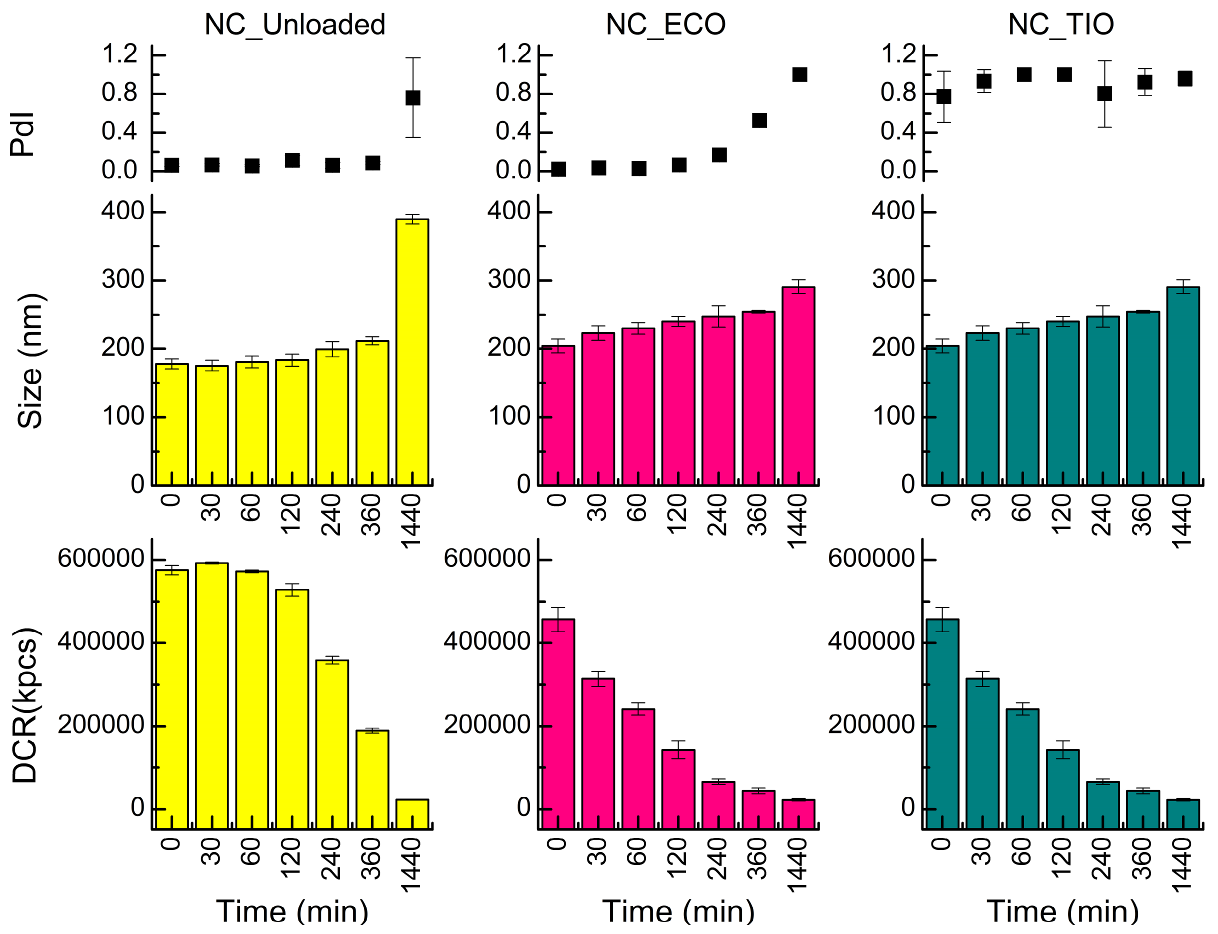

Figure S2. Stability of NCs in DMEM media supplemented with 10% fetal bovine serum, 1% L-glutamine and 1% antibiotic used for MTT assay.

***In vitro* release studies**

The data obtained from *in vitro* release studies were computed using DDsolver [1], which is an Excel-plugin module, and the resultant data were fitted to six different kinetic models [2].

In all models, F is the fraction (%) of released drug in time, t. The adjusted coefficient of determination ($R^2_{adjusted}$, Tables 2 and 3) was estimated for each model, fitted and used as a model ability to describe a given dataset.

1) Zero order

Equation 1: $F = K_0 \times t$

Where K_0 is the zero order release constant.

2) First order

Equation 2: $F = 100 \times (1 - e^{-K_1 \times t})$

Where K_1 is the first order release constant.

3) Higuchi

Equation 3: $F = K_H \times \sqrt{t}$

Where K_H is the Higuchi release constant.

4) Korsmeyer-Peppas

Equation 4: $F = K_{KP} \times t^n$

Where K_{KP} is the release constant incorporating structural and geometric characteristics of the drug-dosage form and n is the diffusional exponent indicating the drug-release mechanism.

5) Hixson-Crowell

Equation 5: $F = 100 \times (1 - (1 - K_{HC} \times t))^3$

Where K_{Hc} is the Hixson-Crowell release constant.

6) Weibull

Equation 6: $F = 100 \times \left(1 - e^{-\frac{(t-Ti)^{\beta}}{\alpha}}\right)$

Where α denotes a scaling parameter that describes the time dependence, T_i accounts for the lag time measured as a result of the dissolution process. While β is the shape parameter which characterizes the curve as either exponential (β=1), sigmoid, S-shaped, with upward curvature followed by a turning point (β>1), or parabolic, with a higher initial slope and after that consistent with the exponential (β<1). T_i is the location parameter, which represents the lag time before the onset of the dissolution or release process and, in most cases, will be near to zero.
